# Factors Associated With Low Birth Weight in Cambodia: A Secondary Analysis of the Cambodia Demographic and Health Survey, 2021–2022

**DOI:** 10.1155/jp/5551643

**Published:** 2026-04-07

**Authors:** Samnang Um, Sothy Heng, Sok Sakha, Leng Dany, Chantrea Sieng, Sovandara Heng

**Affiliations:** ^1^ The National Institute of Public Health, Phnom Penh, Cambodia, szu.cz; ^2^ Management Team, Ministry of Health, Phnom Penh, Cambodia, behdasht.gov.ir; ^3^ The Elite Angkor Clinic, Siem Reap, Cambodia; ^4^ Calmette Hospital, Phnom Penh, Cambodia; ^5^ Khmer Soviet Friendship Hospital, Phnom Penh, Cambodia

**Keywords:** antenatal care, Cambodia Demographic and Health Survey, low birth weight, maternal factors, newborn

## Abstract

**Aims:**

We examined the prevalence of low birth weight (LBW) across provinces and factors associated with LBW among newborns in Cambodia.

**Methods:**

We analyzed the most recent children′s data from the 2021–2022 Cambodia Demographic and Health Survey (CDHS). A total of 4565 weighted newborns were included in the study. Provincial variation in the prevalence of LBW was visualized using ArcGIS. Multiple logistic regression analyses were performed to identify factors associated with LBW.

**Results:**

The prevalence of LBW was 5.9% (95% CI: 5.2–6.8), with the highest rates observed in Ratanak Kiri (13.9%), Kratie (12.8%), Pailin (12.7%), and Svay Rieng (10.2%). After adjusting for confounders, significant factors associated with higher odds of LBW included maternal unemployment (AOR = 1.62; 95% CI: 1.03–2.56), being a first‐born child (AOR = 1.73; 95% CI: 1.26–2.38), and rural area (AOR = 1.41; 95% CI: 1.00–2.01). Attending four or more antenatal care (ANC) visits was protective against LBW (AOR = 0.65; 95% CI: 0.47–0.91). Maternal age and household wealth were not independently associated with LBW in the adjusted model.

**Conclusion:**

The prevalence of LBW among newborns in Cambodia is lower than in Southeast Asia and worldwide. However, significant provincial variations in LBW prevalence exist, particularly in remote areas. Maternal employment, firstborn status, rural residence, and ANC utilization are key determinants of LBW. Public health programs should prioritize provinces with persistently high rates of LBW prevalence and improve ANC coverage, especially for first‐time mothers in rural areas, to further reduce LBW and improve neonatal outcomes.

## 1. Introduction

The World Health Organization (WHO) defines newborn low birth weight (LBW) as a weight below 2500 g (or 5.5 lbs) at birth [1]. LBW is a serious public health issue affecting developed and developing nations [1]. Globally, in 2020, an estimated 19.8 million (or 14.6%) babies suffered from LBW [1, 2]. According to estimates, LBW is more prevalent in South Asia (28%), sub‐Saharan Africa (13%), Latin America (9%), and East Asia and the Pacific (6%) [3, 4]. According to the UNICEF–WHO, the prevalence of LBW in Cambodia was 15.4% in 2000 [4]. Subsequently, the LBW prevalence declined to around 8% of all live births in 2005, remaining stable at about 8% in 2010 and 7.9% in 2014, but it showed a slight decrease to approximately 5.9% in 2021–2022 [5–7]. However, newborns with LBW are more common in rural areas (7.2%) than in urban areas (4%). The highest rates of LBW were seen in remote and low‐income provinces, like Ratanakiri (14.8%), Pailin (12.9%), Mondolkiri (10.7%), and Stung Treng (10.2%), in 2021–2022 [7]. Approximately 2.04% of all deaths were related to LBW in Cambodia [8]. Furthermore, compared with newborns with normal birth weight, those with LBW had a five times higher risk of dying before turning 5 years old [9]. Mortality and morbidity in children are independently associated with newborns with LBW [10, 11]. Additionally, LBW increases the risk of chronic noncommunicable diseases (NCDs) in adulthood, including diabetes and cardiovascular disease [1, 12, 13].

A lack of iron and folic acid (IFA) supplementation and deworming during pregnancy [14], maternal weight gain [15], preterm birth [16], inadequate antenatal care (ANC) visits [17], being anemic [18], being underweight during pregnancy [19], tobacco use [20], and alcohol consumption during pregnancy [21] were associated with an increased risk of LBW. The risk of LBW is 3.8 times higher for mothers younger than 20 years of age or older than 35 years of age compared with mothers 20–29 years old [22].

Mothers with low socioeconomic statuses commonly have LBW babies. For example, LBW newborns were more likely to be delivered by women from the lowest income households (adjusted odds ratio [AOR] = 1.7) and middle‐class households (AOR = 1.6) than mothers from rich households [23]. A previous study in Cambodia indicated that factors independently associated with increased odds of LBW included the mother′s lack of education, with an AOR (AOR = 1.6), and babies being born to mothers with fewer than four ANC visits each during their pregnancies (AOR = 2.0) [17].

The Royal Government of Cambodia (RGC) has recognized that nutrition is the highest priority program in Cambodia, and the Cambodian prime minister has closely monitored it to enforce policy implementation to reach its Sustainable Development Goal [24]. Cambodia′s goal is to reduce the LBW to less than 6% in 2023 and 4% in 2025 [24]. To achieve this goal, the government has implemented various strategies, including counseling during ANC visits; the routine supplementation of IFA for pregnant women; providing nutritious food such as super cereals to pregnant women residing in highly food‐insecure areas; and raising awareness about early pregnancy, smoking, and alcohol consumption during pregnancy [14]. Despite these efforts, the prevalence of LBW remains a significant public health problem in Cambodia. The proportion of LBW in Cambodia slowly decreased in newborns from 2010 to 2022, particularly in remote provinces among vulnerable mothers [5–7]. In addition, LBW has been documented as an independent factor associated with childhood malnutrition [1, 2, 25], infectious disease [1, 4, 26], and the risk of under‐five mortality in Cambodia [1, 4, 9].

To the authors′ knowledge, a limited study using updated data to investigate the association between socioeconomic and maternal factors and LBW has not been explored. A prior study described the prevalence of LBW across provinces in Cambodia, and its associated factors covering the period of 2010–2014 have been published [17]. Therefore, this study was aimed at examining the prevalence of LBW across provinces and the factors associated with LBW among newborns in Cambodia using updated CDHS 2021–2022 data. We hope that this study will facilitate a deeper understanding of the maternal, child, and household socioeconomic factors contributing to LBW among newborns in Cambodia. It may contribute to developing policies and programs incorporating more effective strategies and interventions aimed at reducing LBW prevalence, thereby significantly contributing to the ongoing efforts to decrease newborn mortality in Cambodia.

## 2. Material and Methods

### 2.1. Ethics Statement

The CDHS received approval from the National Ethical Committee for Health Research, Ministry of Health of Cambodia on May 10, 2021 (Ref: 083 NECHR), and the Institutional Review Board (IRB) of ICF International in Rockville, Maryland, United States. The survey data are publicly accessible upon request through the Demographic and Health Surveys (DHS) website (URL: http://dhsprogram.com/data/available-datasets.cfm) [27]. The request must register the user and include information from principal investigators and coinvestigators, a research project title, and a description of the analysis proposed to be performed with the data. Finally, the authorization letter for using data was also obtained from the DHS measure. Before conducting the interview, field researchers explained the survey′s objective to the parents/guardians of each participant under 18 years of age, and written informed consent was obtained from the parents/guardians. The study contains no individual identifiers that could affect the confidentiality of the participants, and the data were used for analysis purposes only.

### 2.2. Data Source

**Figure 1:**
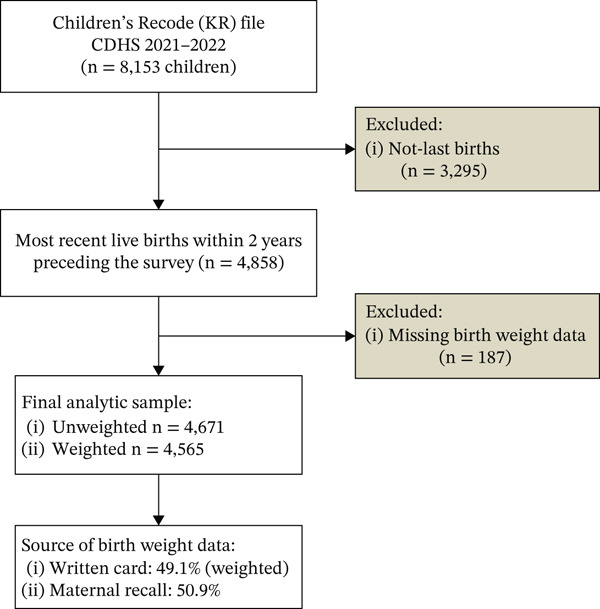
Flowchart of sample selection for the analysis of low birth weight using the CDHS 2021–2022.

This study was a retrospective secondary data analysis using children′s data (KR file) from the most recent CDHS 2021–2022, a nationally representative population‐based household survey from September 15, 2021, to February 15, 2022. The CDHS employed a two‐stage stratified cluster sampling technique to select participants from every province. In the first stage, 709 clusters, or enumeration areas (EAs), were selected (241 EAs from urban areas and 468 EAs from rural areas). In the second stage, a systematic random sample was applied to 25–30 households from each cluster or EA for a total sample size of 21,270 families. Interviews were then conducted with 19,496 women aged 15–49 who had given birth within the 5 years preceding the survey, with a response rate of 98.2%. A total of 8153 children′s information was collected from their mothers [7]. Our analysis was restricted to the last pregnancy, with the most recent live births taking place in the 2 years preceding the survey, and featured reported birth weight information based on either written records, cards, and/or maternal recall [7]. We included these based on the availability of birth weight, ANC utilization variables, place of birth, types of delivery, and maternal health–related factors; CDHS was collected, which provided more reliable birth weight information and reduced the likelihood of introducing recall bias in the study [7]. As a result, we excluded 3295 not‐last births and 187 newborns with missing birth weight data. The final sample included in this analysis was 4671 newborns (4565 weighted counts). Birth weight information was obtained either from written health cards or from maternal recall, in accordance with standard DHS procedures. Among the 4565 weighted newborns included in the analysis, 49.1% of birth weight data were derived from written record cards and 50.9% from maternal recall (Figure 1).

### 2.3. Measurements

#### 2.3.1. Outcome Variable

The newborn′s birth weight was the outcome variable. LBW is defined as a birth weight of less than 2500 g [1]. Then, the original variable was recoded into the binary variable, where non − LBW = 0 (if birth weight ≥ 2500 g) and LBW = 1 (if birth weight < 2500 g).

#### 2.3.2. Right‐Hand Sided Variables

Maternal characteristics included the following:

• Women′s age in years (≤ 19, 20–29, and ≥ 30), marital status (married and not married), and educational attainment (no education/incomplete primary, incomplete primary/incomplete secondary, and completed secondary/higher).
• Employment status (not working, nonprofessional, and professional).
• Having the first ANC visit in the first trimester of pregnancy (yes and no); the number of ANC visits (fewer than four visits and four or more visits); having ANC visits with skilled personnel, such as a medical doctor, nurse, or midwife (yes and no); receiving a tetanus injection (no dose, one dose, and two or more doses); giving iron tablets during pregnancy; intestinal parasite medication (yes and no); and having adequate ANC components. (The defined six interventions included blood pressure measurement, the blood sample collected, the urine sample collected, health and nutrition education, the ultrasound conducted, and counseling on danger signs.)
• Types of deliveries (normal birth and cesarean birth) and delivery locations (home and health facilities).
• Cigarette smoking (nonsmoker and smoker) and alcohol drinking (yes and no).
• Healthcare barriers (no barrier and one or more possible barriers reported, including distance, money, and waiting time) and health insurance coverage (yes and no).

Child characteristics included the following:

• Sex of the child (boy or girl).
• Birth interval (first child, < 2 years, 2–3 years, and 3+).
• Birth order (1, 2–3, and 4+).

Socioeconomic characteristics included the following:

• Households′ wealth index (poorest, poorer, middle, richer, and richest). The wealth index was calculated via principal component analysis (PCA) using variables for household assets and dwelling characteristics [7], the drinking water source, the toilet facility type (improved or unimproved), and the place of residence (rural and urban).
• Cambodia′s provinces were regrouped for analytic purposes into a categorical variable with five geographical regions: Phnom Penh capital city, the plains, the Tonle Sap, the coastal/sea, and the mountains [28].

## 3. Analytic Strategy

We followed the methods of Chhea et al. [17] to analyze LBW′s prevalence across provinces and factors associated with LBW among newborns in Cambodia. Statistical analyses were performed using STATA Version 18 (StataCorp 2021). We formally incorporated the DHS′s complex sample design using the “survey” package; all estimations were carried out using the svy command in our descriptive and logistic regression analyses. Data evaluation and cleaning followed standard procedures for ensuring data quality [29]. Descriptive statistics for the maternal, child, and household socioeconomic characteristics were described using weighted frequency distributions. The provincial variation in the prevalence of LBW was done using ArcGIS software Version 10.8 [30]. A shapefile for Cambodian administrative boundaries was obtained from the United Nations for the Coordination of Humanitarian Affairs (OCHA) (URL: http://humdata.org/dataset/cod-ab-khm; license: http://humdata.org/faqs/licenses). Bivariate analysis using chi‐square tests was performed to assess associations between independent variables (maternal, child, and household socioeconomic characteristics) and LBW. The final multiple logistic regression analyses included variables that were associated with LBW at a *p* value ≤ 0.10 [25] or that had potential confounder variables, including maternal age at the time of giving birth [22]. Simple logistic regression determined the magnitude of associations between LBW and maternal, child, and household socioeconomic characteristics. The results were reported as odds ratios (ORs) with 95% confidence intervals (CIs). Multiple logistic regressions were then used to assess the independent factors associated with LBW after adjusting for other potential confounding factors in the model. The results from the final adjusted model were reported as AORs with 95% CIs and corresponding *p* values.

Multicollinearity between the independent variables was checked before the final regression model was fit; these variables included maternal age at the time of giving birth, educational level, employment status, number of ANC visits during pregnancy, child′s birth order, wealth index, and place of residence [31]. Model diagnostics included assessment of goodness‐of‐fit using the Hosmer–Lemeshow test, visual inspection of predicted probabilities of LBW, and evaluation of model selection criteria, including the Akaike information criterion (AIC) and Bayesian information criterion (BIC). Potential interaction terms, such as maternal age by ANC visits, birth order by ANC visits, and maternal age by wealth index, were tested for statistical significance using joint Wald tests.

## 4. Results

Approximately 70% of the mothers were aged 20–29 at childbirth, with 39% having no schooling or incomplete primary education, 46.6% completing primary or incomplete secondary education, and 14.5% completing secondary or higher education. Around 31% were not employed, and almost 95% were married (Table 1). Only 1.1% of the mothers smoked cigarettes, whereas 36.3% reported drinking alcohol in the past few months. Most mothers (86.3%) attended at least four ANC visits each during pregnancy. Likewise, 87.8% initiated ANC visits within the first trimester of pregnancy, and 98.7% attended them with skilled providers. Most mothers (89.1%) consumed iron supplements, whereas 82.3% took antiparasite medication during pregnancy. About 60.7% of the mothers received adequate ANC components. Approximately 24.8% of mothers had health insurance. Hospital deliveries accounted for 96.8% of all births. Among these births, more than half were boys, and singleton births constituted 99.5%. Among the sample, 99.1% had live births. Their average birth weight was 3088 g. A total of 32.4% of newborns were first‐order births, and 10.2% were fourth‐ or higher order births. About 40.2% of newborns were born into low‐wealth index households, and 60.8% were born in rural areas.

**Table 1:** Distribution of newborns by background characteristics, CDHS 2021–2022 (*N* = 4565 weighted count).

Variables	Total births	%
Maternal characteristics		
Age at the time of giving birth		
≤ 19	1083	23.7
20–29	3145	68.9
≥ 30	338	7.4
Educational attainment		
No schooling/incomplete primary	1776	38.9
Complete primary/incomplete secondary	2128	46.6
Complete secondary/higher	661	14.5
Employment status		
Not working	1395	30.6
Professional	1273	27.9
Nonprofessional	1897	41.6
Marital status		
Not married	231	5.1
Married	4334	94.9
Smoking cigarettes		
Nonsmoker	4515	98.9
Smoker	50	1.1
Alcohol intake in the past month		
No	2910	63.7
Yes	1655	36.3
Number of ANC visits during pregnancy		
< 4 visits	625	13.7
≥ 4 visits	3940	86.3
First ANC visit in the first trimester		
No	501	11.0
Yes	4007	87.8
Antenatal care from a skilled provider		
Unskilled	61	1.3
Skilled	4504	98.7
Tetanus toxoid injections during pregnancy		
None	767	16.8
One dose	666	14.6
Two or more	3133	68.6
Took iron supplementation during pregnancy		
No	499	10.9
Yes	4067	89.1
Took antiparasite medication during pregnancy		
No	806	17.7
Yes	3759	82.3
Compliance with ANC components		0.0
Inadequate	1797	39.4
Adequate	2769	60.7
Healthcare barriers		
No barrier	1869	40.9
One or more barriers	2696	59.1
Health insurance coverage		
No	3432	75.2
Yes	1133	24.8
Child′s characteristics		
Place of birth		
Home	147	3.2
Health facilities	4418	96.8
Sex of child		
Girl	2233	48.9
Boy	2332	51.1
Multiple births		
Single	4544	99.5
Twin	22	0.5
Child alive		
Alive	4525	99.1
Death	40	0.9
Child′s weight at birth		
Mean (SD)	3088 g (SD = 9.8)	
< 2500 g	272	5.9
> 2500 g	4294	94.1
Child′s birth interval (year)		
First child	1479	32.4
< 2 years	305	6.7
2–3 years	500	11.0
Three or more	2281	50.0
Child′s birth order		
1st child	1479	32.4
2–3 child	2619	57.4
Four or more	467	10.2
Type of delivery		
Normal delivery	3717	81.4
Cesarean section	848	18.6
Household characteristics		
Wealth index		
Poor	1835	40.2
Middle	847	18.6
Rich	1883	41.2
Type of toilet facility		
Improved	4000	87.6
Nonimproved	565	12.4
Source of drinking water		
Improved	2747	60.2
Unimproved	1818	39.8
Place of residence		
Urban	1789	39.2
Rural	2776	60.8
Geographic region		
Phnom Penh		
Plain	683	15.0
Tonle Sap	1556	34.1
Coastal	1408	30.8
Plateau/mountain	270	5.9

*Note:* Survey weights are applied to obtain weighted percentages. Phnom Penh capital city; plains: Kampong Cham, Tboung Khmum, Kandal, Prey Veng, Svay Rieng, and Takeo; Tonle Sap: Banteay Meanchey, Kampong Chhnang, Kampong Thom, Pursat, Siem Reap, Battambang, Pailin, and Otdar Meanchey; coastal/sea: Kampot, Kep, Preah Sihanouk, and Koh Kong; and mountains: Kampong Speu, Kratie, Preah Vihear, Stung Treng, Mondul Kiri, and Ratanak Kiri.

**Figure 2:**
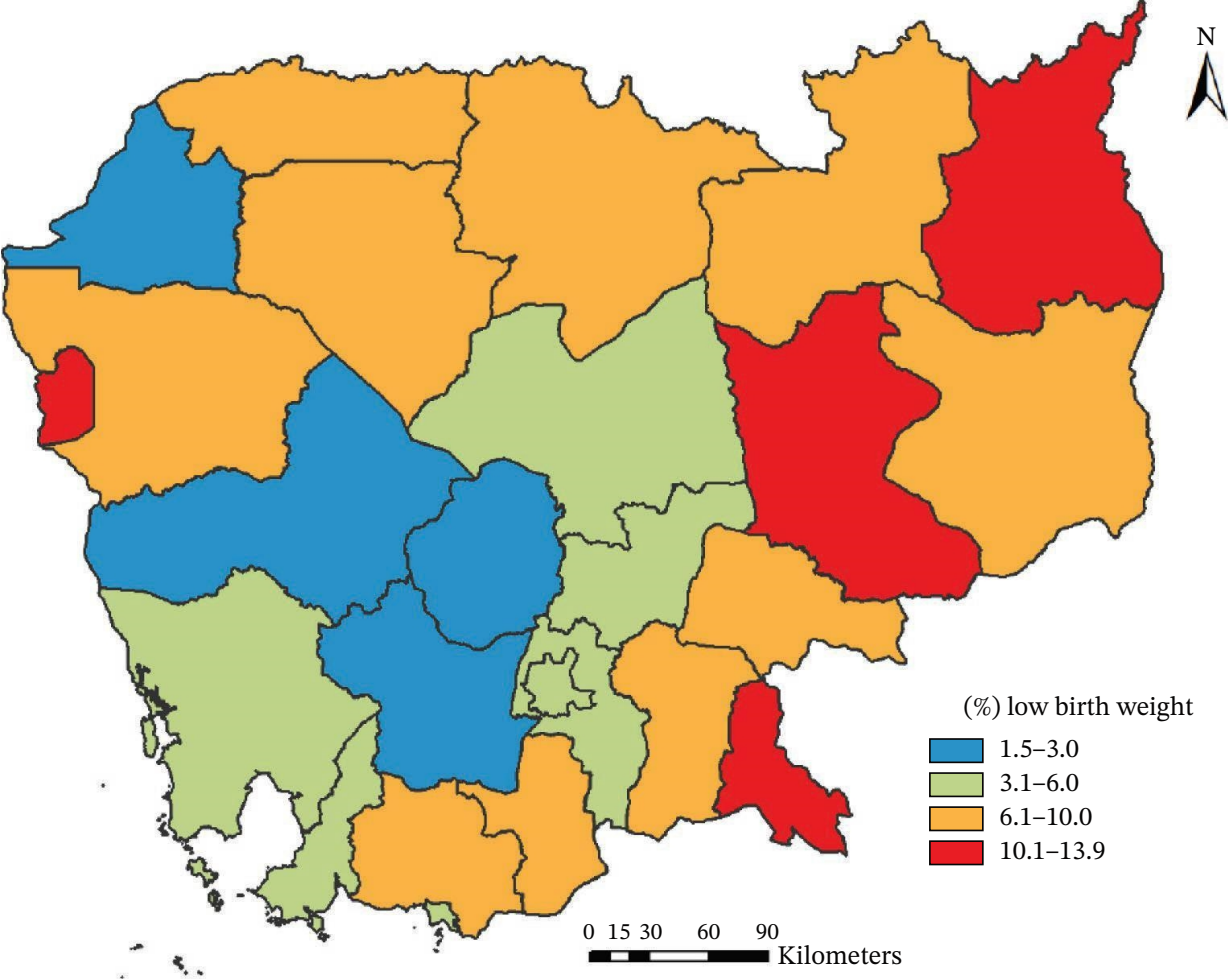
Prevalence of newborns with low birth weight by province. The map was created using ArcGIS software Version 10.8 [30]. A shapefile for Cambodian administrative boundaries was obtained from the United Nations for the Coordination of Humanitarian Affairs (OCHA) (http://humdata.org/dataset/cod-ab-khm; license: http://humdata.org/faqs/licenses).

The prevalence of LBW was 5.9% (95% CI: 5.2–6.8). Among newborns, the highest rates of LBW were observed in Ratanak Kiri (13.9%), Kratie (12.8%), Pailin (12.7%), Svay Rieng (10.2%), Stung Treng (9.2%), and Tboung Khmum (8.3%). Conversely, the lowest rates were recorded in Kampong Speu (1.5%), Banteay Meanchey (2.5%), Pursat (2.6%), and Kampong Chhnang (2.7%) (Figure 2).

### 4.1. Factors Associated With LBW Among the Most Recent Live Births

In the bivariate analysis (Table 2), several factors were significantly associated with the prevalence of LBW in Cambodia. Healthcare utilization was a critical factor; mothers who attended fewer than four ANC visits during pregnancy had a significantly higher prevalence of LBW infants (8.6%) than those who attended four or more visits (5.5%, *p* = 0.007). Child characteristics also played a major role, specifically birth order (*p* = 0.004) and birth interval (*p* = 0.012). First‐born children had a higher LBW prevalence (7.9%) compared to second‐ and third‐born children (4.8%). Socioeconomic and geographic factors further influenced outcomes, with newborns from poor households (7.1%) significantly more affected by LBW than those from rich households (4.8%, *p* = 0.046). Similarly, infants born in rural areas had a significantly higher prevalence of LBW (6.9%) compared to those in urban settings (4.6%, *p* = 0.005).

**Table 2:** Distribution of sociodemographic factors and proportion of newborns with LBW in Cambodia, 2021–2022 (*N* = 4565 weighted count).

Variables	Low birth weight (*N* = 272)			Normal weight (*N* = 4294)			*p* value
	Freq.	%	95% CI	Freq.	%	95% CI	
Maternal characteristics							
Age at the time of giving birth							
≤ 19	71	6.5	(5.1–8.4)	1012	93.5	(91.6–94.9)	0.484
20–29	176	5.6	(4.7–6.7)	2969	94.4	(93.3–95.3)	
≥ 30	25	7.5	(4.1–13.2)	313	92.5	(86.8–95.9)	
Educational attainment							
No schooling/incomplete primary	109	6.2	(4.9–7.8)	1667	93.8	(92.2–95.1)	0.869
Complete primary/incomplete secondary	127	5.9	(4.9–7.3)	2001	94.1	(92.7–95.1)	
Complete secondary/higher	36	5.4	(3.4–8.5)	626	94.6	(91.5–96.6)	
Employment status							
Not working	103	7.4	(5.9–9.3)	1292	92.6	(90.7–94.1)	**0.014**
Professional	53	4.2	(3.0–5.7)	1220	95.8	(94.3–97.0)	
Nonprofessional	116	6.1	(5.0–7.5)	1781	93.9	(92.5–95.0)	
Marital status							
Not married	17	7.4	(4.4–12.0)	214	92.6	(88.0–95.6)	0.387
Married	255	5.9	(5.1–6.7)	4080	94.1	(93.3–94.9)	
Alcohol intake in the past month							
No	171	5.9	(4.9–7.0)	2739	94.1	(93.0–95.1)	0.832
Yes	100	6.1	(4.9–7.5)	1555	93.9	(92.5–95.1)	
Number of ANC visits during pregnancy							
< 4 visits	54	8.6	(6.5–11.3)	571	91.4	(88.7–93.5)	**0.007**
≥ 4 visits	218	5.5	(4.7–6.4)	3722	94.5	(93.6–95.3)	
First ANC visit in the first trimester							
No	36	7.2	(5.0–10.3)	465	92.8	(89.7–95.0)	0.294
Yes	233	5.8	(5.0–6.7)	3774	94.2	(93.3–95.0)	
Tetanus toxoid injections during pregnancy							
None	43	5.6	(4.0–7.9)	724	94.4	(92.1–96.0)	0.929
One dose	41	6.1	(4.4–8.4)	625	93.9	(91.6–95.6)	
Two or more	188	6.0	(5.1–7.1)	2945	94.0	(92.9–94.9)	
Took iron supplementation during pregnancy							
No	33	6.7	(4.7–9.4)	466	93.3	(90.6–95.3)	0.500
Yes	238	5.9	(5.1–6.7)	3828	94.1	(93.3–94.9)	
Took antiparasite medication during pregnancy							
No	52	6.5	(4.6–9.1)	754	93.5	(90.9–95.4)	0.583
Yes	219	5.8	(5.0–6.7)	3540	94.2	(93.3–95.0)	
Compliance with ANC components							
Inadequate	110	6.1	(5.0–7.5)	1687	93.9	(92.5–95.0)	0.745
Adequate	162	5.8	(4.9–7.0)	2607	94.2	(93.0–95.1)	
Healthcare barriers							
No barrier	101	5.4	(4.2–6.8)	1768	94.6	(93.2–95.8)	0.277
One or more barriers	171	6.3	(5.4–7.5)	2525	93.7	(92.5–94.6)	
Health insurance coverage							
No	216	6.3	(5.4–7.3)	3216	93.7	(92.7–94.6)	0.141
Yes	55	4.9	(3.6–6.6)	1078	95.1	(93.4–96.4)	
Place of birth							
Home	13	8.7	(4.8–15.1)	134	91.3	(84.9–95.2)	0.195
Health facilities	259	5.9	(5.1–6.7)	4159	94.1	(93.3–94.9)	
Child′s characteristics							
Sex of child							
Female	145	6.5	(5.3–7.9)	2088	93.5	(92.1–94.7)	0.247
Male	127	5.4	(4.4–6.7)	2206	94.6	(93.3–95.6)	
Child′s birth interval (year)							
First child	117	7.9	(6.4–9.8)	1362	92.1	(90.2–93.6)	**0.012**
< 2 years	15	5.1	(2.8–8.9)	290	94.9	(91.1–97.2)	
2–3 years	26	5.2	(3.4–7.7)	474	94.8	(92.3–96.6)	
Three or more	113	5.0	(4.0–6.1)	2168	95.0	(93.9–96.0)	
Child′s birth order							
1st child	117	7.9	(6.4–9.8)	1362	92.1	(90.2–93.6)	**0.004**
2–3 child	124	4.8	(3.9–5.8)	2495	95.2	(94.2–96.1)	
Four or more	30	6.4	(4.2–9.6)	437	93.6	(90.4–95.8)	
Type of delivery							
Normal delivery	220	5.9	(5.1–6.8)	3497	94.1	(93.2–94.9)	0.883
Cesarean section	52	6.1	(4.2–8.7)	797	93.9	(91.3–95.8)	
Household characteristics							
Wealth index							
Poor	130	7.1	(5.8–8.6)	1706	92.9	(91.4–94.2)	**0.046**
Middle	52	6.1	(4.6–8.1)	796	93.9	(91.9–95.4)	
Rich	90	4.8	(3.7–6.2)	1793	95.2	(93.8–96.3)	
Type of toilet facility							
Improved	229	5.7	(5.0–6.6)	3771	94.3	(93.4–95.0)	0.104
Nonimproved	43	7.5	(5.5–10.2)	523	92.5	(89.8–94.5)	
Source of drinking water							
Improved	165	6.0	(5.0–7.2)	2582	94.0	(92.8–95.0)	0.844
Unimproved	106	5.8	(4.7–7.2)	1712	94.2	(92.8–95.3)	
Place of residence							
Urban	80	4.5	(3.4–5.8)	1709	95.5	(94.2–96.6)	**0.005**
Rural	192	6.9	(6.0–8.0)	2584	93.1	(92.0–94.0)	
Geographic region							
Phnom Penh	26	3.8	(2.0–7.0)	657	96.2	(93.0–98.0)	
Plain	108	6.9	(5.6–8.6)	1448	93.1	(91.4–94.4)	0.138
Tonle Sap	77	5.5	(4.3–6.9)	1331	94.5	(93.1–95.7)	
Coastal	16	5.9	(3.7–9.1)	254	94.1	(90.9–96.3)	
Plateau/mountain	45	6.9	(5.4–8.8)	604	93.1	(91.2–94.6)	

*Note:* Survey weights are applied to obtain weighted percentages. The bold values indicate statistically significant results (*p* < 0.05).

Conversely, several variables did not show statistically significant associations with LBW at this level. These included maternal age (*p* = 0.484), educational attainment (*p* = 0.869), alcohol intake (*p* = 0.832), and iron supplementation (*p* = 0.500). Furthermore, no significant variations were found regarding the sex of the child, place of birth, or the timing of the first ANC visit, suggesting that while these factors are important for general maternal health, they were not primary drivers of birth weight variation in this specific sample (Table 2).

### 4.2. Factors Associated With LBW Among the Most Recent Live Births in a Multiple Logistic Regression

In the multivariable logistic regression analysis (Table 3), several maternal, child, and household factors remained independently associated with increased odds of LBW. The model included maternal age at the time of giving birth, maternal employment status, ANC visits, birth order, household wealth index, and place of residence. After adjusting for potential confounders, maternal employment status emerged as a significant predictor; newborns of mothers who were not working had 1.62 times higher odds of being LBW compared to those born to mothers in professional occupations (AOR = 1.62; 95% CI: 1.03–2.56).

**Table 3:** Unadjusted and adjusted factors associated with LBW among the most recent live births in Cambodia, CDHS 2021–2022.

Variables	Total births (*N* = 4565)		Total births (*N* = 4565)	
	Unadjusted OR	95% CI	Adjusted AOR	95% CI
Maternal characteristics				
Age at the time of giving birth				
≤ 19	1.18	(0.84–1.66)	1.06	(0.75–1.49)
20–29	Ref.		Ref.	
≥ 30	1.36	(0.68–2.72)	1.30	(0.68–2.56)
Educational attainment				
No schooling/incomplete primary	1.04	(0.73–1.47)		
Complete primary/incomplete secondary	Ref.			
Complete secondary/higher	0.90	(0.51–1.57)		
Employment status				
Not working	**1.84** ^∗∗∗^	**(1.20–2.82)**	**1.62** ^∗∗^	**(1.03–2.56)**
Professional	Ref.		Ref.	
Nonprofessional	**1.50** ^∗∗^	**(1.01–2.21)**	1.37	(0.89–2.09)
Marital status				
Not married	Ref.			
Married	0.78	(0.45–1.36)		
Alcohol intake in the past month				
No	Ref.			
Yes	1.03	(0.77–1.39)		
Number of ANC visits during pregnancy				
< 4 visits	Ref.		Ref.	
≥ 4 visits	**0.62** ^∗∗∗^	**(0.44–0.88)**	**0.67** ^∗∗^	**(0.48–0.93)**
First ANC visit in the first trimester				
No	Ref.			
Yes	0.80	(0.52–1.22)		
Tetanus toxoid injections during pregnancy				
None	Ref.			
One dose	1.10	(0.66–1.82)		
Two or more	1.07	(0.71–1.61)		
Took iron supplementation during pregnancy				
No	Ref.			
Yes	0.87	(0.58–1.30)		
Took antiparasite medication during pregnancy				
No	Ref.			
Yes	0.89	(0.59–1.34)		
Compliance with ANC components				
Inadequate	Ref.			
Adequate	0.95	(0.71–1.28)		
Healthcare barriers				
No barrier	Ref.			
One or more barriers	1.19	(0.87–1.63)		
Health insurance coverage				
No	Ref.			
Yes	0.76	(0.53–1.10)		
Place of birth				
Home	Ref.			
Health facilities	0.65	(0.34–1.25)		
Child′s characteristics				
Sex of child				
Female	Ref.			
Male	0.83	(0.60–1.14)		
Child′s birth interval (year)				
First child	Ref.			
< 2 years	0.62	(0.32–1.18)		
2–3 years	**0.63** ^∗^	**(0.39–1.03)**		
Three or more	**0.60** ^∗∗∗^	**(0.43–0.85)**		
Child′s birth order				
1st child	**1.73** ^∗∗∗^	**(1.24–2.40)**	**1.73** ^∗∗∗^	**(1.26–2.38)**
2–3 child	Ref.		Ref.	
Four or more	1.36	(0.82–2.27)	1.20	(0.73–1.98)
Type of delivery				
Normal delivery	Ref.			
Cesarean section	1.03	(0.67–1.58)		
Household characteristics				
Wealth index				
Poor	**1.51** ^∗∗^	**(1.07–2.13)**	1.06	(0.66–1.59)
Middle	1.29	(0.85–1.95)	1.02	(0.71–1.58)
Rich	Ref.		Ref.	
Type of toilet facility				
Improved	Ref.			
Nonimproved	1.34	(0.94–1.91)		
Source of drinking water				
Improved	Ref.			
Unimproved	0.97	(0.71–1.32)		
Place of residence				
Urban	Ref.		Ref.	
Rural	**1.59** ^∗∗∗^	**(1.15–2.21)**	**1.41** ^∗∗^	**(1.00–2.01)**
Geographic region				
Phnom Penh	Ref.			
Plain	1.90 ^∗^	(0.96–3.78)		
Tonle Sap	1.48	(0.74–2.95)		
Coastal	1.59	(0.71–3.54)		
Plateau/mountain	1.88 ^∗^	(0.94–3.78)		

*Note:* Survey weights are applied to obtain weighted percentages. The bold values indicate statistically significant results (*p* < 0.05).

Abbreviation: Ref, reference value.

^∗∗∗^ *p* < 0.01,  ^∗∗^ *p* < 0.05, and  ^∗^ *p* < 0.1.

Healthcare utilization was a critical protective factor: Mothers who attended four or more ANC visits during their last pregnancy had 33% lower odds of having an LBW newborn compared to those who attended fewer than four visits (AOR = 0.67; 95% CI: 0.48–0.93).

Regarding child characteristics, being a first‐born child remained significantly associated with LBW, carrying 1.73 times higher odds compared to second‐born children (AOR = 1.73; 95% CI: 1.26–2.38).

Places of residence factors also significantly influenced outcomes, with newborns in rural areas having 1.41 times higher odds of LBW than urban newborns (AOR = 1.41; 95% CI: 1.00–2.01).

Notably, after adjusting for these variables in the multivariable model, maternal age and the household wealth index were not statistically significant predictors of LBW. Specifically, while the “poor” wealth index was significant in the unadjusted model (OR = 1.51), this association disappeared after controlling for other factors (AOR = 1.06; 95% CI: 0.66–1.59).

Model diagnostics indicated a good fit and moderate discrimination. The Hosmer–Lemeshow goodness‐of‐fit test suggested no evidence of lack‐of‐fit (*F* (9651) = 1.53; *p* = 0.132), and the area under the receiver operating characteristic curve (AUC) was 0.60 (95% CI: 0.56–0.63), indicating moderate ability of the model to distinguish between LBW and non‐LBW cases. Variance inflation factors were all below 2, suggesting no evidence of multicollinearity. We also assessed interaction terms between maternal age and ANC visits, birth order and ANC visits, and maternal age and wealth. Interaction analyses showed that maternal age × ANC visits was not significant (*F* (7653) = 1.43; *p* = 0.190), birth order × ANC visits was significant overall (*F* (5655) = 3.22; *p* = 0.007), and maternal age × wealth was significant overall (*F* (11,649) = 2.07; *p* = 0.021). However, individual interaction terms were unstable with wide CIs and were therefore not retained in the final model to preserve interpretability and parsimony.

## 5. Discussion

The prevalence of LBW among newborns in Cambodia has declined notably, reaching 5.9% (95% CI: 5.2–6.8) compared with the previous rates of 11% in 2000, 8% in 2005, and 7.9% in 2010 and 2014 [5, 6, 32]. This decline is particularly striking compared with South Asia, where the prevalence was 24.9%, and the global prevalence of 14% in 2020 [33]. The reduction aligns with Cambodia′s efforts to reduce the mortality of neonates, infants, and children under five [7] and may reflect the government′s investments in health infrastructure, particularly in rural areas, including the expansion of health facilities, provision of essential medical equipment and supplies, an increase in the number of midwives, and improved access to skilled care during pregnancy and childbirth [24, 34, 35]. Additionally, increased access to maternal health services has contributed significantly to improved birth outcomes [36]. According to CDHS 2021–2022, institutional births increased from 19.3% to 98%, mothers attending four or more ANC visits increased from 9% to 86.1%, and mothers initiating ANC visits in the first trimester increased from 10% to 87% between 2000 and 2022 [7]. In the same years, 98% of women took iron tablets or syrup, and 84% took intestinal parasite medications [7]. The government has also encouraged early and routine ANC visits through a monetary incentive of $20 for each of up to four ANC visits at any health facility with a contract with the National Social Security Fund [37, 38].

The variation in the prevalence of LBW among newborns is highest in low‐economic provinces, such as Ratanak Kiri, Kratie, Pailin, Svay Rieng, and Stung Treng (Figure 1). Limited implementation and enforcement of maternal and child health policies in remote areas likely contribute to these disparities [14].

This study identified several factors independently associated with LBW in Cambodia. Newborns of mothers with primary education, those born to unemployed mothers, first‐born children, and children born in rural areas had higher odds of LBW. Conversely, mothers attending at least four ANC visits during their last pregnancy were less likely to have LBW infants.

Maternal employment status was a significant determinant, with newborns of mothers who were not working having 1.62 times higher odds of being LBW compared with those born to mothers in professional occupations. This finding aligns with previous studies suggesting that maternal employment provides financial resources, greater access to healthcare, and improved nutritional status, all of which reduce the risk of LBW [39, 40].

Mothers who attended four or more ANC visits during their last pregnancy had 33% lower odds of delivering an LBW infant compared with those who attended fewer than four visits. This supports the WHO and Cambodia Ministry of Health recommendations for at least four ANC visits, which enable regular monitoring, nutritional counseling, laboratory assessments, tetanus immunization, and early identification of complications [35].

Being a first‐born child was associated with 1.73 times higher odds of LBW compared with second‐born children, consistent with prior research in Cambodia and other low‐ and middle‐income countries; the first child has a higher likelihood of having LBW than the second or third child [17, 40, 41].

The study found that children born in mothers′ residences in rural areas were 1.41 times more likely to have LBW. This association is consistent with previous studies [42]. According to research conducted in multiple developing nations, a rural residence is a risk factor for LBW newborns [41]. This may be due, in part, to the limited implementation and enforcement of these policies and interventions in the country′s remote areas. Rural areas have higher poverty rates and restricted access to ANC services than urban areas [43, 44].

Interestingly, maternal age and household wealth were not independently associated with LBW in the adjusted model. While the “poor” wealth category was significant in unadjusted analyses (OR = 1.51), this association disappeared after controlling for other factors (AOR = 1.06; 95% CI: 0.66–1.59), suggesting that variables such as ANC utilization and maternal employment may mediate the relationship between socioeconomic status and LBW.

Our study has several strengths. First, this study used the most recent data from a nationally representative household survey with a high response rate of 97%. Data were collected using validated survey methods and highly trained data collectors, which contributed to improved data quality. Second, the analysis was restricted to the last newborns with the most recent live births in the 2 years preceding the survey to minimize maternal recalled birth weight information, contributing to knowledge of LBW prevalence and associated factors in Cambodia. Third, the DHS complex survey design and sampling weights were incorporated into the analysis, and a multiple logistic regression model was used to adjust for potential confounding. Model diagnostics indicated good fit (Hosmer–Lemeshow *p* = 0.132), moderate discrimination (AUC = 0.60), and no multicollinearity (VIF < 2), supporting the robustness and reliability of the findings.

However, it is essential to acknowledge the study′s several significant limitations. First, due to the cross‐sectional data collected in the CDHS 2021–2022, the study was confined to establishing associations between variables rather than causality. The available data were obtained from household surveys and not during pregnancy or at the time of birth. Although many variables, such as maternal age at birth, household wealth index, and place of residence, are unlikely to have changed, others, such as maternal employment status and marital status, may have changed between the pregnancy or delivery and the survey. Another issue encountered was handling missing data regarding the outcome variable for LBW. Nevertheless, this issue is relatively minor, as less than 1% of the data was found to be missing in the CDHS 2021–2022 dataset. Second, the study was restricted to variables for which the CDHS 2021–2022 collected data. Many potential risk factors or contributing factors to LBW were not well documented. These included maternal malnutritional status (including anemia and weight gain during pregnancy); HIV infection; young married age; LBW; history of preterm births; and morbidity and illnesses during pregnancy, including NCDs, like hypertension and diabetes.

In conclusion, the prevalence of LBW in Cambodia continues to decline but remains unevenly distributed across provinces. Independent factors associated with LBW include maternal employment status, ANC attendance, birth order, and rural residence. These findings support the current national policy promoting four or more ANC visits during pregnancy and highlight the need for targeted interventions in rural and low‐resource areas, as well as strategies to improve maternal education and employment opportunities.

**Nomenclature**
ANC: antenatal care
AOR: adjusted odds ratio
CDHS: Cambodia Demographic Health Survey
EAs: enumeration areas
IFA: iron and folic acid
LBW: low birth weight
NCDs: noncommunicable diseases
NECHR: National Ethical Committee for Health Research
NSSF: National Social Security Fund
RGC: Royal Government of Cambodia
PPS: probability proportional to size
WHO: World Health Organization

## Funding

No funding was received for this manuscript.

## Disclosure

This manuscript was previously published as a preprint on medRxiv: 10.1101/2024.02.25.24303351v1.

## Conflicts of Interest

The authors declare no conflicts of interest.

## Data Availability

The data that support the findings of this study are available from the corresponding author upon reasonable request.
